# Structure and mechanical properties of high-weight-bearing and low-weight-bearing areas of hip cartilage at the micro- and nano-levels

**DOI:** 10.1186/s12891-020-03468-y

**Published:** 2020-07-02

**Authors:** Jiang-Bo Guo, Ting Liang, Yan-Jun Che, Hui-Lin Yang, Zong-Ping Luo

**Affiliations:** 1 Department of Orthopaedics, the First Affiliated Hospital of Soochow University, Suzhou, Jiangsu 215006 People’s Republic of China; 2 Department of Orthopaedics, Orthopaedic Institute, the First Affiliated Hospital, Soochow University, Suzhou, Jiangsu 215006 People’s Republic of China

**Keywords:** Cartilage, Collagen fiber, Elastic modulus, Micro-scale, Nano-scale, Weight-bearing

## Abstract

**Background:**

Articular cartilage has a high-weight-bearing area and a low-weight-bearing area, the macroscopic elastic moduli of the two regions are different. Chondrocytes are affected by the applied force at the microscopic level. Currently, the modulus of the two areas at the micro and nano levels is unknown, and studies on the relationship between macro-, micro- and nano-scale elastic moduli are limited. Such information may be important for further understanding of cartilage mechanics. Moreover, the surface morphology, proteoglycan content, and micro and nano structure of the two areas, which influences the mechanical properties of cartilage should be discussed.

**Methods:**

Safranin-O/Fast Green staining was used to evaluate the surface morphology and semi-quantify proteoglycan content of porcine femoral head cartilage between the two weight-bearing areas. The unconfined compression test was used to determine the macro elastic modulus. Atomic force microscope was used to measure the micro and nano compressive elastic modulus as well as the nano structure. Scanning electron microscope was employed to evaluate the micro structure.

**Results:**

No significant differences in the fibrillation index were observed between two areas (*P* = 0.5512). The Safranin-O index of the high-weight-bearing area was significantly higher than that of the low-weight-bearing area (*P* = 0.0387). The compressive elastic modulus of the high-weight-bearing area at the macro and micro level was significantly higher than that of the low-weight-bearing area (*P* = 0.0411 for macro-scale, and *P* = 0.0001 for micro-scale), while no statistically significant differences were observed in the elastic modulus of collagen fibrils at the nano level (*P* = 0.8544). The density of the collagen fibers was significantly lower in the high-weight-bearing area (*P* = 0.0177). No significant differences were observed in the structure and diameter of the collagen fibers between the two areas (*P* = 0.7361).

**Conclusions:**

A higher proteoglycan content correlated with a higher compressive elastic modulus of the high-weight-bearing area at the micro level than that of the low-weight-bearing area, which was consistent with the trend observed from the macroscopic compressive elastic modulus. The weight-bearing level was not associated with the elastic modulus of individual collagen fibers and the diameter at the nano level. The micro structure of cartilage may influence the macro- and micro-scale elastic modulus.

## Background

Articular cartilage is an important weight-bearing structure of the human body. In particular, the hip joint is the comparatively large weight-bearing joint that plays an important role in joint lubrication, joint friction reduction, and pressure damping [1]. Articular cartilage consists of a small number of chondrocytes and a large number of extracellular matrices. Chondrocytes maintain the normal metabolism of the cartilage. The extracellular matrix mainly contains type II collagen, proteoglycans, and water, and the extracellular matrix content and the cross-linking mechanism of collagen affect normal functioning of cartilage [2]. Articular cartilage tissue is subject to long-term cyclic loads, and cartilage is closely related to the load it carries. For example, osteoarthritis is closely related to stress, since long-term exposure to excessive stress can cause osteoarthritis. An important method in the conservative treatment of osteoarthritis is to relieve the weight load of the joint [3].

Regarding articular cartilage, weight-bearing is non-uniform and spatially specific. Based on the different cartilage locations, the femoral head cartilage area can be easily divided into a high-weight-bearing area (HWA) and a low-weight-bearing area (LWA) [4, 5]. The relationship between HWA and LWA is very close. For example, cartilage with HWA subjected to long-term cyclic stress is prone to develop degenerative damages, such as osteoarthritis, and transplantation of autologous LWA cartilage at the damaged HWA has been used in the clinic to repair joint injury [6–8]. Regarding the design of tissue material for cartilage repair, it is necessary to consider the magnitude of cartilage load under normal conditions, as well as the difference in mechanical properties between different weight-bearing areas. Both parameters are critical for the design of future cartilage substitute materials [9, 10]. Macro-mechanical studies have shown that different loading areas of cartilage have different mechanical properties [11–15], but the chondrocytes are affected at the microscopic level by the applied force [16]. From a microscopic point of view, the chondrocytes are very sensitive to microscopic forces and the surrounding micro-nano structures, and mechanical properties alter with the load changes in the surrounding microscopic environment [17–19]. Therefore, the mechanical properties of collagen fibers in the HWA and the LWA at the micro-nano level, and the relationship between macro and micro-nano levels are of utmost importance for further understanding of the cartilage mechanical properties. Moreover, the mechanical properties of cartilage are mainly related to the cross-linking mechanism of collagen fibers and proteoglycan content [20], and such significant factors need to be discussed.

Given the aforementioned micromechanical properties, and because the micro level is relatively close to the macroscopic level, it was assumed that the elastic modulus at the micro level should be consistent with the macroscopic compressive elastic modulus trend of the cartilage. For nano-scale mechanical properties, individual fibers are measured, therefore, the differences between the two regions may be minor. At the micro level, the micro structure of cartilage in the HWA should be more conducive to load-bearing. However, the differences in structure at the nano-scale are less obvious due to the smaller range of the detection region. The proteoglycan content should be consistent with the trend of elastic modulus between two areas. During physiological function, cartilage is a load-bearing component, and under compression conditions, its mechanical properties are consistent with the physiological conditions of the body. Therefore, an atomic force microscope (AFM) mechanical test was performed to measure the micro- and nano-scale compressive elastic modulus and the nano-scale structure of cartilage. In addition, scanning electron microscope (SEM) was employed to scan and evaluate the structure of micron-sized cartilage. Safranin-O/Fast Green staining was performed to evaluate the surface morphology and to semi-quantify proteoglycan content of the cartilage.

## Materials and methods

### Sample preparation

**Fig. 1:**
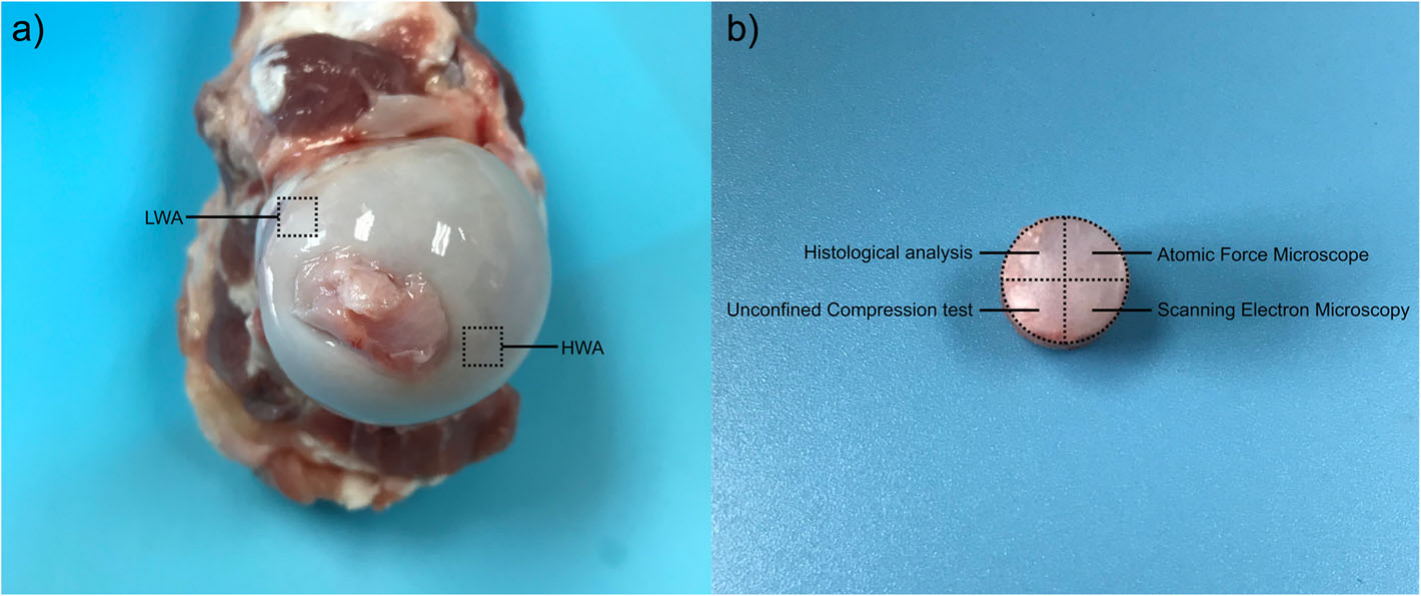
Sample preparation. (**a**) High-weight-bearing area (HWA) and Low-weight-bearing area (LWA) of porcine femoral head cartilage (**b**) Experimental design

In this study, healthy femoral head cartilage from 8-month-old porcine (*n* = 6) without degeneration was used. Fresh healthy porcine femoral heads that were slaughtered the day before, were purchased from the same local malls (Yangcanli Market, Jiangsu Province, China) in the morning. No significant differences in the weight and volume of the porcine femoral heads were observed. To expose the femoral head cartilage, muscles and ligaments were sheared and cleaned. The weight-bearing condition of the cartilage of the femoral head of porcine is similar to that of humans, and two different weight-bearing zones were marked (Fig. 1a), including the HWA above the femoral head, and the LWA below the femoral head [4, 5]. The entire cartilage layer was harvested using an electric drill connected with a broken nail extractor (diameter = 8 mm), then divided into four specimens using surgical blades for subsequent experiments, each specimen corresponded to a specific experiment (Fig. 1b). Specimens for histological analysis were fixed in 10% neutral buffered formalin (Shanghai Yuanye Bio-Technology Co Ltd., Shanghai, China) for 24 h, whereas other specimens stored in a refrigerator at − 20 °C for later use [21]. All animal experiments were strictly performed under the guidelines of the Chinese Council for Animal Care, approved by the Animal Care Committee of the Laboratory Animal at School of Medicine, SooChow University.

### Histological analysis

HWA and LWA specimens were fixed in 10% neutral buffered formalin (Shanghai Yuanye Bio-Technology Co Ltd., Shanghai, China) for 24 h. After gradient alcohol (Sinopharm Chemical Reagent Co Ltd., Shanghai, China) dehydration, specimens were paraffin-embedded (Leica, Richmond, VA, USA) for serial sectioning using a histotome (Leica, Richmond, VA, USA) at a thickness of 6 μm. Safranin O/Fast Green staining was performed on the cartilage tissue sections according to the kit instructions (Beijing Solarbio Science & Technology Co Ltd., Beijing, China). Staining was evaluated using a binocular microscope (XSP-2CA, Shanghai, China). Two histologic sections of each sample were used to obtain the fibrillation index (FI) to determine the cartilage surface morphology at a magnification of 100 × as previously described [22]. Per sample, two sections were used for the Safranin-O index to semi-quantify the cartilage proteoglycan content in the top 20 μm at a magnification of 200 × as previously described [23].

### Macro elastic modulus of cartilage obtained by the unconfined compression test

Within 24 h after specimen removal, an unconfined compression test to 60% strain was performed at a displacement rate of 0.6 mm/min using a biomechanical testing machine (Shanghai Heng Wing Precision Instrument Co Ltd., Shanghai, China) and a 50 N mechanical sensor. Each sample (*n* = 6 per area) was tested once. The macro elastic modulus, the slope of the stress strain curve, was calculated using the linear region of the curve in Origin 8.0 (Northampton, MA, USA) [24, 25].

### Micro-nano elastic modulus measurement and nano-sized collagen fiber morphological observation by AFM

Within 24 h after specimen removal, the cartilage specimen was embedded in Optimal Cutting Temperature (OCT) compound, and frozen sections were cut to get the top layer of the cartilage (thickness = 20 μm) using a freezing microtome (Leica, Richmond, VA, USA). Sections were adhered to a glass slide, and biomechanical analysis was performed using an AFM scanner (Dimension ICON, Bruker, USA). To obtain the micro-scale elastic modulus, at the micrometer scale, twelve locations per stress zone were randomly pressed using a spherical tip at a diameter of 5 μm. At the nano scale, fifteen collagen fibrils were randomly selected per area to measure the nano-biomechanical property using a ScanAsyst-Air probe at a radius of 5 nm. The cartilage nano-scale topography was also scanned at the nanometer level. Fifteen collagen fibers were randomly selected per area from cartilage nano images, and the diameter of collagen fibers was determined by using the scale tool in Adobe Photoshop CS5 (Adobe Systems Incorporated, San Jose, CA, USA) [26].

### Morphological observation of micron-sized collagen fibers by SEM

The cartilage samples were fixed in 2.5% glutaraldehyde for 4 h at 4 °C, then washed in PBS 0.01 M for 2 h. Then, samples were processed by the NaOH cell-maceration method [27, 28]. In brief, samples were immersed in 10% NaOH solution for 3–4 days at room temperature, then rinsed in distilled water for 3 days. Next, samples were immersed in 1% tannic acid for 2 h. Subsequently, samples were dehydrated with 70, 80, 90, and 100% alcohol for 4 h, dried in a critical point dryer (Leica, Richmond, VA, USA) with liquid CO_2_, and coated with ion sputter (Quorum Technologies, East Sussex, UK). The collagen cross-linking mechanism including the direction of collagen fibers alignment, spacing and their differences along the HWA and the LWA were observed by SEM (Quanta 250, FEI, Hillsboro, Oregon, USA) at a magnification of 1000 ×. The density of collagen fibers was calculated in fifteen regions of each weight-bearing area at a magnification of 3000 ×.

### Statistical analysis

Data from all experiments are presented as the means ± standard deviation or median (interquartile range, IQR) as appropriate. Data management and statistical analysis were performed using Microsoft Excel 2016 (Microsoft Corp., Edmond, WA, USA) and GraphPad Prism 8.0.2 (GraphPad Software Inc., San Diego, CA, USA). Significant differences in FI, Safranin-O index, macro-scale elastic modulus, and the density of collagen fiber between two groups were obtained by using the Mann-Whitney test, and significant differences in the micro- and nano-scale elastic modulus and the diameter of collagen fibers between two groups were obtained by using the unpaired t-test. Statistical significance was set at *p* ≤ 0.05.

## Results

### Histological analysis

**Fig. 2:**
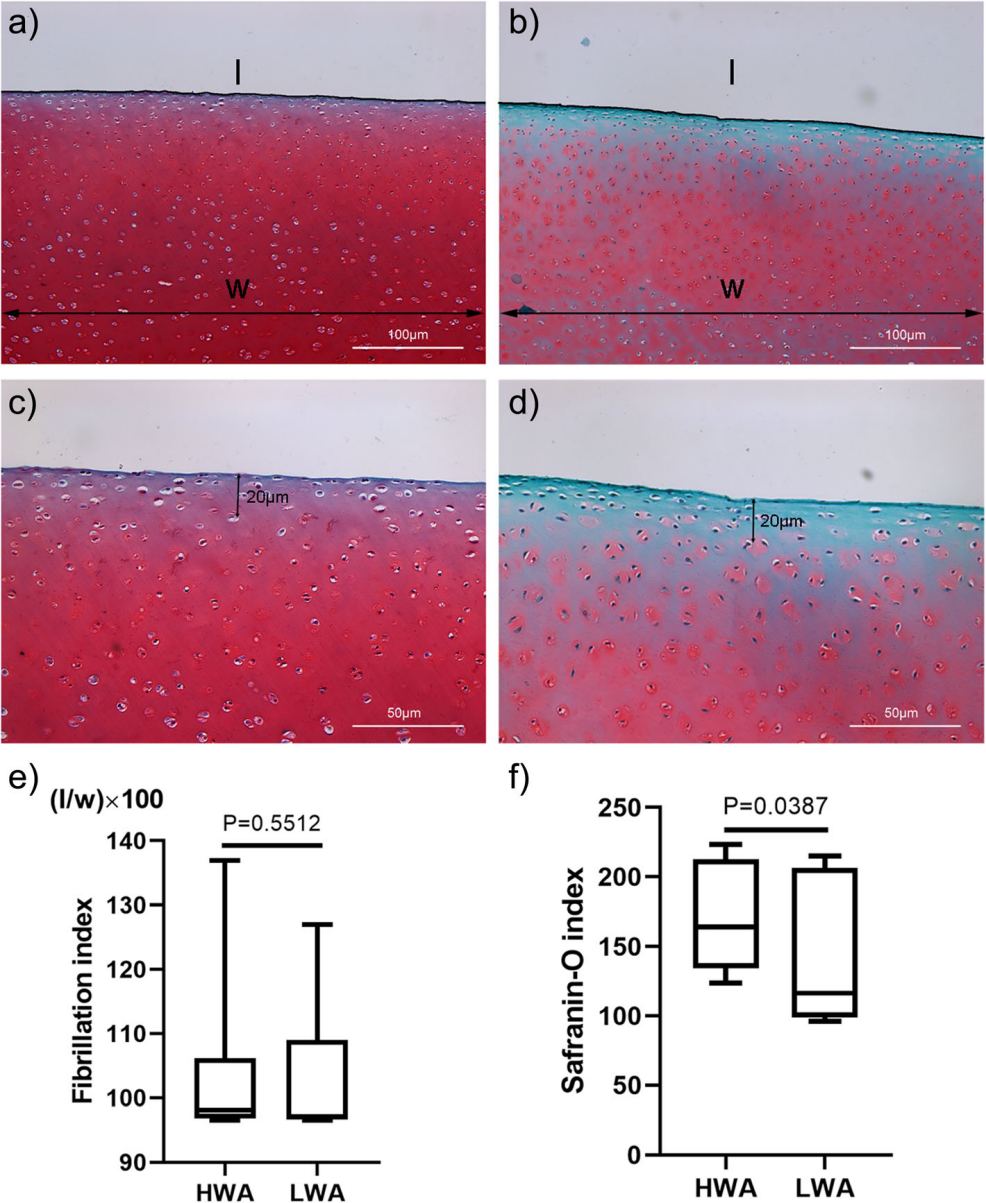
Representative Safranin-O staining images of two areas. (**a**) High-weight-bearing area (HWA) at a magnification of 100 ×. (**b**) Low-weight-bearing area (LWA) at a magnification of 100 ×. (**c**) HWA at a magnification of 200 ×. (**d**) LWA at a magnification of 200 ×. (**e**) Fibrillation index (FI) results of two areas. (**f**). Safranin-O index results of two areas (*n* = 6 per area)

Representative staining results are presented in Fig. 2. Figure 2a and c represented HWA, while 2b and 2d represented LWA. The median FI of HWA was 98.10 (IQR 97.03–105.67), and the median FI of LWA was 97.13 (IQR 96.74–106.31). No statistically significant differences in FI were observed between the two areas (Fig. 2e, *P* = 0.5512). The top 20 μm of LWA showed Safranin-O staining loss as indicated in Fig. 2b and d. The median Safranin-O index of HWA was 164.10 (IQR 138.70–211.02), while the median Safranin-O index of LWA was 116.30 (IQR 102.53–204.70). The Safranin-O index of HWA was significantly higher when compared to that of LWA (Fig. 2f, *P* = 0.0387).

### Femoral head cartilage macro-, micro- and nano-scale elastic modulus

**Fig. 3:**
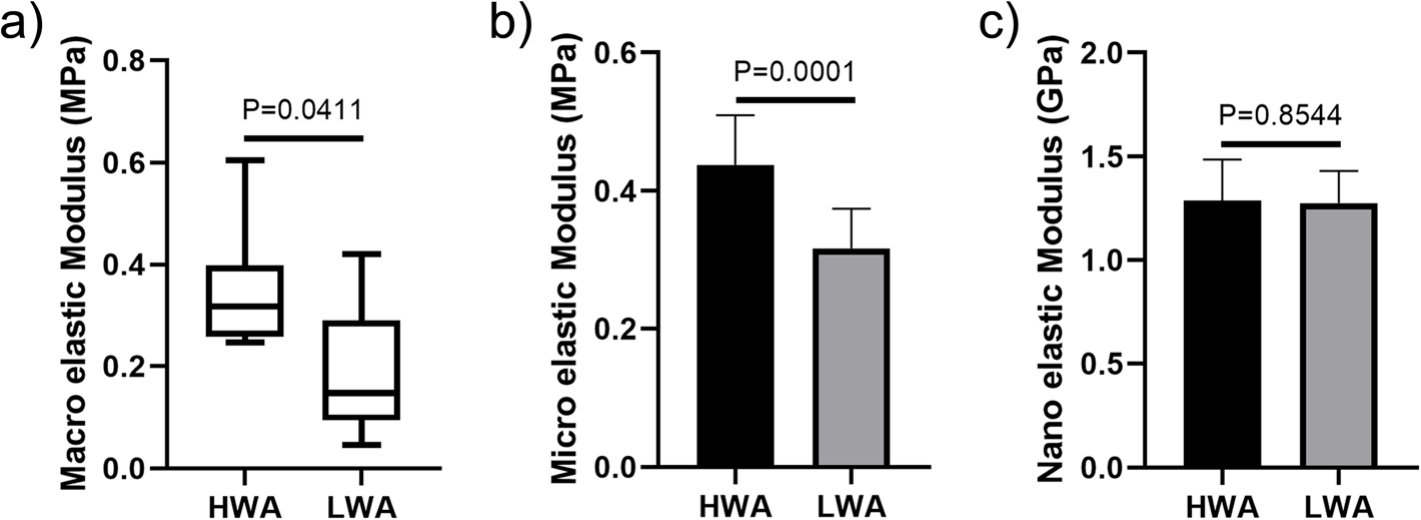
Macro-, micro- and nano-scale elastic modulus of high-weight-bearing area (HWA) and low-weight-bearing area (LWA). (**a**) Macro-scale elastic modulus results. (**b**) Micro-scale elastic modulus results. (**c**) Nano-scale elastic modulus results (*n* = 6 per area)

The median macro-scale elastic modulus of the HWA was 0.32 (IQR 0.27–0.33) MPa, while the median macro-scale elastic modulus of the LWA was 0.15 (IQR 0.12–0.22) MPa. Our data showed that the macro-scale elastic modulus of the femoral head cartilage was significantly higher in the HWA when compared to the LWA (Fig. 3a, *P* = 0.0411). The micro-scale elastic modulus of the HWA was (0.44 ± 0.07) MPa, while the micro-scale elastic modulus of the LWA was (0.32 ± 0.06) MPa. In addition, the micro-scale elastic modulus of the femoral head cartilage was significantly higher in the HWA when compared to the LWA (Fig. 3b, *P* = 0.0001). The nano-scale elastic modulus of the HWA collagen fibers was (1.29 ± 0.20) GPa, while the nano-scale elastic modulus of the LWA collagen fibers was (1.28 ± 0.15) GPa. No statistically significant differences were observed in the elastic modulus of collagen fibril at nano-scale between the two regions (Fig. 3c, *P* = 0.8544).

### Femoral head cartilage nanostructure

**Fig. 4:**
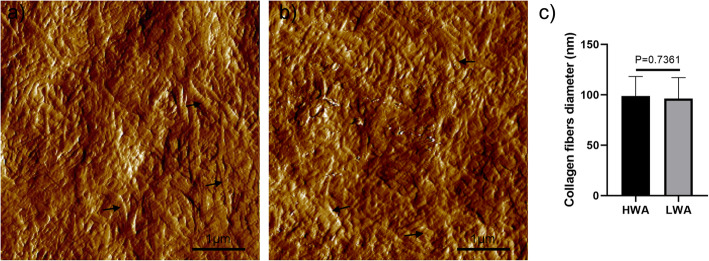
Representative image of the atomic force microscope (AFM) nano-scale morphology of cartilage of two areas. (**a**) High-weight-bearing area (HWA) nano-scale cartilage morphology. (**b**) Low-weight-bearing area (LWA) nano-scale cartilage morphology. (**c**) Diameter of collagen fibers of two areas (*n* = 6 per area)

A representative image of the nanostructure of cartilage is presented in the Fig. 4. At the nano-scale, the arrangement and alignment of collagen fibers in the two weight-bearing areas tended to be randomly distributed. There are no specific rules to follow. The diameter of cartilage collagen fibers in the HWA was (98.73 ± 19.50) nm, while the diameter of collagen fibers in the LWA was (96.21 ± 20.91) nm. No statistically significant differences were observed in the diameter of collagen fibrils at the nano-scale level between the two regions (Fig. 4c, *P* = 0.7361). Furthermore, no significant differences were observed between the nanoscale morphology of the cartilage surface of the HWA and the LWA on the AFM scans.

### Femoral head cartilage microstructure

**Fig. 5:**
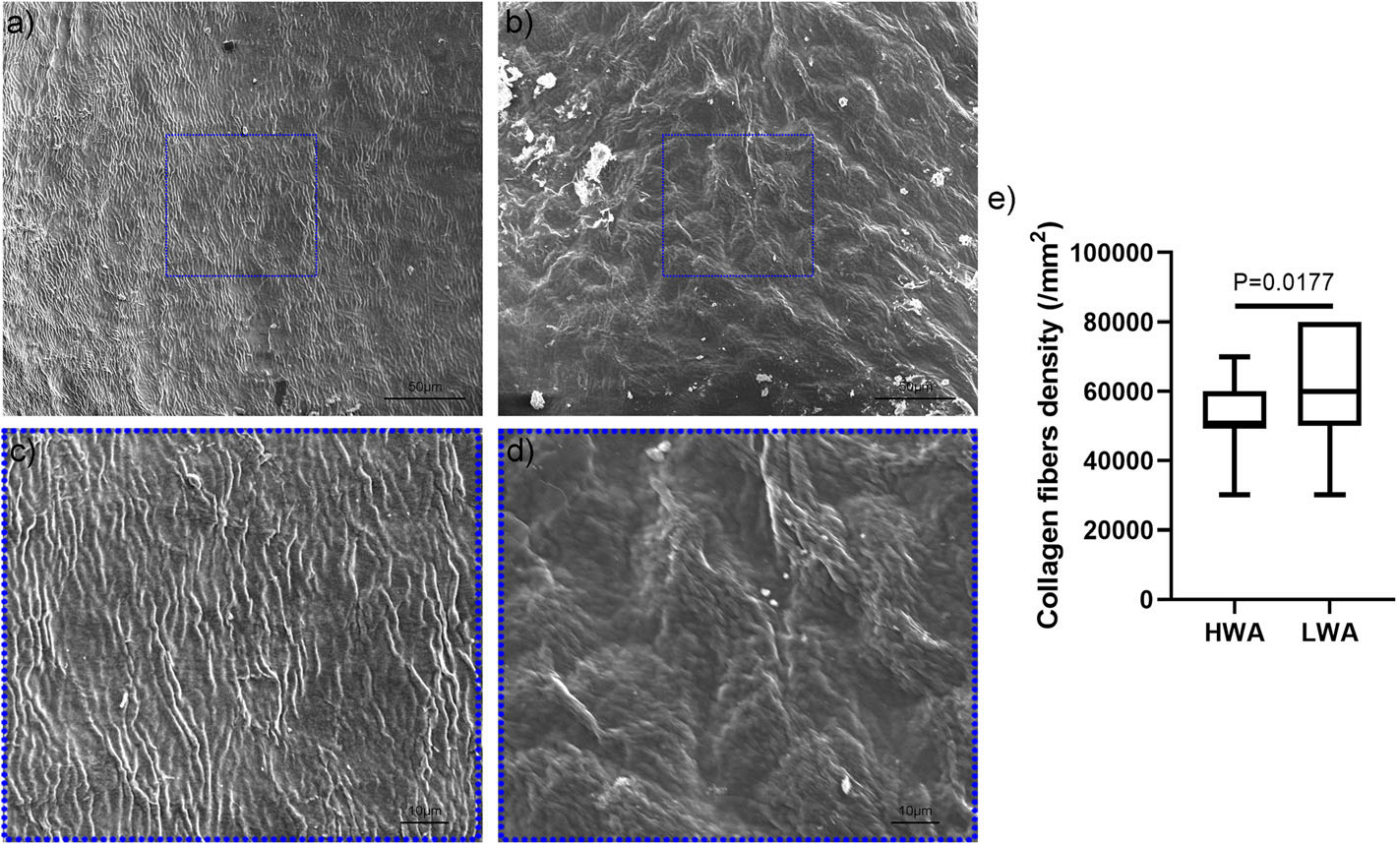
Representative scanning electron microscope (SEM) micro-scale image of the cartilage morphology of the two areas. (**a**) High-weight-bearing area (HWA) at a magnification of 1000 ×. (**b**) Low-weight-bearing area (LWA) at a magnification of 1000 ×. (**c**) HWA at a magnification of 3000 ×. (**d**) LWA at a magnification of 3000 ×. (**e**) The collagen fiber density of the two areas (*n* = 6 per area)

The SEM results at 1000x magnification (Fig. 5a and b) showed that, although the collagen fiber network tended to be randomly arranged in the two weight-bearing areas, both areas have some collagen fibers arranged in a radial direction. The arrangement of collagen fibers and collagen fibers in the HWA is more organized and regular when compared to that in the LWA (Fig. 5a). In the LWA, several collagen fibers tended to aggregate, and spacing of collagen fibers in the LWA was not as well arranged when compared to that in the HWA (Fig. 5b). Calculation of the density of collagen fibers at 3000x magnification (Fig. 5c and d) showed that the median density of collagen fibers in the HWA was 50,000.00 (IQR 50000.00–55,000.00) / mm^2^, and the median density of collagen fibers in the LWA was 60,000.00 (IQR 55000.00–75,000.00) / mm^2^. Thus, when compared to the LWA, the density of collagen fibers in the HWA was significantly lower (*P* = 0.0177). Taken together, our data showed that the collagen fibers in the HWA were better arranged than the collagen fibers in the LWA.

## Discussion

There are differences in macroscopic mechanical properties between the LWA and the HWA of cartilage [11–15]. Chondrocytes are inductive to mechanical effects at the microscopic level, therefore, it is important to know the microscopic mechanical properties and their relationship to macroscopic mechanical properties. Our findings showed that cartilage compressive modulus between HWA and LWA at the micro level was consistent with the trend observed at the macro level, while no significant differences in compressive modulus, diameter and structure of collagen fibers were found at the nano level between two areas. This information is critical to investigate the mechanical properties of cartilage on different load bearing areas. Furthermore, such information is crucial for the development of engineering technology of cartilage tissue. Insight into the micro and nano level of cartilage may provide novel insight for the development of cartilage replacement materials, and may serve as a reference for future research on the micro mechanical properties of osteoarthritis.

The low FI value as determined by our histological studies and the fact that no statistical differences were observed between the two groups indicated that we successfully established normal cartilage of the two areas [22]. The extracellular matrix of cartilage is mainly composed of collagen, proteoglycans, and water. The Safranin-O index was significantly higher in the HWA, thereby indicating that the HWA contained a higher proteoglycan content. These findings were consistent with the data presented in previous studies, in which was reported that a compressive modulus strongly and positively correlated with proteoglycan content [29, 30].

Regarding the macroscopic view of the influence of different weight-bearing level on the elastic modulus of articular cartilage, Shaw et al. reported the effect of different weight-bearing level on the tensile modulus of the knee joint of healthy individuals [11]. Their results demonstrated that the tensile modulus of the LWA was higher when compared to that of the HWA. However, Swann et al. reported that the level of cartilage stiffness was higher in the HWA in the knee and ankle joints [15]. Kempson et al. reported that the stiffest cartilage was located in a band, which extended from the superior surface of the femoral head, up to the anterior and posterior aspects, which indicated that the HWA had a higher modulus [14]. Furthermore, Athanasiou et al. reported that the superior portion of the femoral head cartilage has a higher modulus when compared to the inferior portion of the femoral head cartilage [12]. Karchner et al. reported a higher modulus in the HWA of the stifle joint [13]. The results mentioned above are similar to our macro-level results except for Shaw. The differences between Shaw and others may be attributed to the following, firstly, in macro mechanics, the experimental methods are different. The results from Shaw were measured by tensile testing using isometric tensile apparatus, whereas the results from others were measured by compression testing using indentation machines. Secondly, in previously published studies, the specimens were processed by freezing and thawing before being measured, and the freeze-thaw process may affect the mechanical properties of the specimen and introduce changes [31]. The number of freeze-thaw cycles should be kept to a minimum. Our study was the first to reveal differences in mechanical properties of different weight-bearing areas of normal femoral head cartilage at the micro and nano level, which complements the macro mechanical properties. Our findings showed that HWA cartilage maintained a high elastic modulus at the micro level. The elastic modulus of a single collagen fiber was measured at the nanometer level, and no significant differences were observed in the elastic modulus of collagen fibers between the two weight-bearing areas. The weight-bearing level did not affect the single collagen fiber at the nanometer level, which suggested that the mechanical properties of cartilage at the macro- and micro-scale were affected by multiple factors, not just the stiffness of the collagen fiber [26].

The arrangement of collagen fibers mainly forms the elastic scaffold of the cartilage, and water absorption of the proteoglycans results in local elastic tension and osmotic tension [20]. The collagen cross-linking mechanism, proteoglycans, and water influenced by a different weight-bearing level caused significant differences in the elastic modulus at the macro- and micro-scale [19, 20, 32]. At the micro level, the distribution of collagen fibers in the HWA was more uniform when compared to that in the LWA. In addition, observation of the morphology of cartilage at the nano-scale level, including cross-linking mode and diameter of collagen fibers, revealed no significant differences between the two areas. This may be related to the daily weight-bearing activity in the HWA, which may result in a more organized collagen fiber arrangement and reconstruction to provide sufficient bearing capacity [19, 20, 33]. This reconstruction was only reflected in the cartilage structure at the micro level, whereas the load has not yet affected the structure of collagen fibers at the nano-scale.

The present study has a number of inevitable limitations. Several differences were observed between in vitro-treated samples and direct in vivo detection. Because specimens for the unconfined compression test, SEM, and AFM need preparation, direct in vivo mechanical property measurements are impossible. In this study, we aimed for proper selection of animal specimens. Differences in walking patterns between animals and humans result in different cartilage stress conditions between the two species. However, the animal cartilage model has many advantages. Firstly, it is easier to control the integrity of porcine cartilage. As the human body grows, degeneration of the cartilage tissue occurs with age. The quality of porcine cartilage can be controlled by selecting young and healthy pigs without apparent degeneration, which are then used for experimental studies. Secondly, due to the similarities in cartilage structure and the vascular system between porcine and humans, porcine tissue has been widely used as a mechanical model for research [19, 32, 34, 35]. Because of the slight differences between human and porcine tissue, the porcine femoral head was used for investigating cartilage stress, thus the experiments closely resemble the human condition [36].

## Conclusions

The results confirmed that a higher proteoglycan content in the HWA correlated with a higher micro compressive modulus in the HWA. Data at the micro-scale level was consistent with the trend observed by the macro compressive modulus. Moreover, the micro structure of cartilage may influence the macro- and micro-scale elastic modulus. However, weight-bearing was not associated with single collagen fibers at the nano level, and no significant differences were observed in the compressive modulus, structure, and diameter of the collagen fibers in the two areas. The above findings may provide novel insight for the development of cartilage replacement materials, studies on micromechanical properties of pathologically degenerated cartilage, among others.

## Data Availability

The datasets used and/or analyzed during the current study are available from the corresponding author on reasonable request.

## Abbreviations

HWA: High-weight-bearing area
LWA: Low-weight-bearing area
FI: Fibrillation index
AFM: Atomic force microscope
SEM: Scanning electron microscope
